# Meis1 regulates Foxn4 expression during retinal progenitor cell differentiation

**DOI:** 10.1242/bio.20132279

**Published:** 2013-09-06

**Authors:** Mohammed M. Islam, Ying Li, Huijun Luo, Mengqing Xiang, Li Cai

**Affiliations:** 1 Department of Biomedical Engineering, Rutgers University, 599 Taylor Road, Piscataway, NJ 08854, USA; 2 Center for Advanced Biotechnology and Medicine and Department of Pediatrics, Rutgers University – Robert Wood Johnson Medical School, Piscataway, NJ 08854, USA; 3 State Key Laboratory of Ophthalmology, Zhongshan Ophthalmic Center, Sun Yat-Sen University, 54 Xianlie Road, Guangzhou 510060, China; * Present address: Department of Molecular Biology, UT Southwestern Medical Center, Dallas, TX 75390, USA; ‡ Present address: Department of Biochemistry and Molecular Biology, Mayo Clinic Arizona, Scottsdale, AZ 85259, USA

**Keywords:** Enhancer, Foxn4, Meis1, Retinal progenitor, Horizontal cell, Amacrine cell

## Abstract

The transcription factor forkhead box N4 (Foxn4) is a key regulator in a variety of biological processes during development. In particular, Foxn4 plays an essential role in the genesis of horizontal and amacrine neurons from neural progenitors in the vertebrate retina. Although the functions of Foxn4 have been well established, the transcriptional regulation of *Foxn4* expression during progenitor cell differentiation remains unclear. Here, we report that an evolutionarily conserved 129 bp noncoding DNA fragment (Foxn4CR4.2 or CR4.2), located ∼26 kb upstream of *Foxn4* transcription start site, functions as a *cis*-element for Foxn4 regulation. CR4.2 directs gene expression in Foxn4-positive cells, primarily in progenitors, differentiating horizontal and amacrine cells. We further determined that the gene regulatory activity of CR4.2 is modulated by Meis1 binding motif, which is bound and activated by Meis1 transcription factor. Deletion of the Meis1 binding motif or knockdown of Meis1 expression abolishes the gene regulatory activity of CR4.2. In addition, knockdown of Meis1 expression diminishes the endogenous *Foxn4* expression and affects cell lineage development. Together, we demonstrate that CR4.2 and its interacting Meis1 transcription factor play important roles in regulating *Foxn4* expression during early retinogenesis. These findings provide new insights into molecular mechanisms that govern gene regulation in retinal progenitors and specific cell lineage development.

## Introduction

The vertebrate retina is an excellent model to study the development of the nervous system including the cell differentiation process. Although more than 50 subtypes of retinal neurons have been identified (Masland, 2001), the vertebrate retina is mainly composed of six major types of neurons and one major type of glial cells. These seven major cell types are derived from a common pool of multipotent retinal progenitor cells (RPC) that differentiate in a conserved chronological order (Livesey and Cepko, 2001). Retinal ganglion cells, cone photoreceptors, horizontal and amacrine cells are produced first, whereas rod photoreceptors, Müller glial cells and bipolar cells are generated last. The RPC differentiation pathway is determined by both cell-intrinsic (e.g. transcription factors) and cell-extrinsic factors (e.g. growth factors). Many transcription factors have been found to regulate the genesis and/or differentiation of one or more retinal cell types (Hatakeyama et al., 2001; Liu et al., 2001; Inoue et al., 2002; Yang et al., 2003; Li et al., 2004; Fujitani et al., 2006). An excellent way to gain an understanding of how these factors work together in networks is the dissection of gene regulatory elements of key transcription factors.

Forkhead box N4 transcription factor (Foxn4) plays an essential role in vertebrate retinal development (Gouge et al., 2001; Li et al., 2004; Boije et al., 2013). In mice, chicken and lower vertebrates like fish and tadpole (*Xenopus laevis*), the gene is expressed in brain tissue, spinal cord, olfactory organs, lung and the retina (Gouge et al., 2001; Danilova et al., 2004; Kelly et al., 2007; Boije et al., 2008; Li and Xiang, 2011). *Foxn4* is also expressed in the atrioventricular canal (Chi et al., 2008) and in the thymus (Schorpp et al., 2002; Danilova et al., 2004) of adult zebrafish. In the developing chicken retina, Foxn4 expression starts around embryonic day 3 (E3 or Hamburger–Hamilton stage 18, HH18) and ends around E8.5 (HH35) (Li et al., 2004; Boije et al., 2008). Foxn4 controls the genesis of horizontal and amacrine cells which are interneurons that modulate and integrate visual signals in the retina and are born early from multipotent RPCs (Li et al., 2004; Liu et al., 2013). Furthermore, the loss of *Foxn4* completely abolishes the horizontal cell and causes a switch in the cell fate to rod photoreceptor cells (Li et al., 2004). Although its essential functions during tissue development have been well established, little is known about the molecular mechanisms that regulate the spatiotemporal expression of *Foxn4*.

Meis1 is a member of TALE (Three Amino acid Loop Extension) homeodomain transcription factors involved in many processes of vertebrate development and morphogenesis, e.g. maintaining RPC status, regulating the expression of key retinal developmental genes and retinal development in vertebrate species (Heine et al., 2008; Erickson et al., 2010). Meis1 specifies positional information in the retina and tectum to organize the zebrafish visual system (Erickson et al., 2010). Meis1 marks RPCs throughout the period of neurogenesis in the retina (Heine et al., 2008). In addition, loss of Meis1 expression causes impaired retinal progenitor cell proliferation (Heine et al., 2008) as well as partial ventralization of the retina (Erickson et al., 2010). Although many studies have demonstrated the essential role of Meis1 protein in retinal development, its downstream target genes and detailed mechanisms of how it functions in RPCs and retinal development remain largely uncharacterized.

Comparative genomic analysis has been demonstrated as a successful method to identify evolutionarily conserved regulatory elements that direct cell/tissue-specific gene expression (Marshall et al., 1994; Aparicio et al., 1995; de la Calle-Mustienes et al., 2005; Fisher et al., 2006; Prabhakar et al., 2006; Pennacchio et al., 2007; Emerson and Cepko, 2011). Highly conserved noncoding sequences are extensively associated with spatiotemporal and quantitative regulation of gene expression, development and disease (Kleinjan and van Heyningen, 2005; Davidson and Erwin, 2006). Genome comparisons using the human, mouse, chicken and other vertebrate sequences reveal remarkable conservation of the *Foxn4* gene.

To identify regulatory elements involved in the transcriptional regulation of *Foxn4* expression in the retina, we assessed four evolutionarily conserved noncoding DNA sequences using a reporter assay system with the aid of *in ovo* electroporation technique (Doh et al., 2010; Islam et al., 2012). A highly conserved region with 129 bp noncoding sequence (Foxn4CR4.2 or CR4.2) was shown to direct gene expression preferentially in horizontal and amacrine cells. The activity of CR4.2 is regulated by Meis1 transcription factor as demonstrated by electrophoretic mobility shift assay (EMSA) and site-directed mutagenesis assay. Furthermore, knockdown of *Meis1* using a short hairpin RNA (shRNA) gene silencing method diminishes the gene regulatory activity of CR4.2 and severely affects *Foxn4* expression. These findings shed new light on the regulatory mechanism of Foxn4 expression during retinal cell differentiation.

## Results

### Identification of cis-elements at the Foxn4 locus

**Fig. 1.**
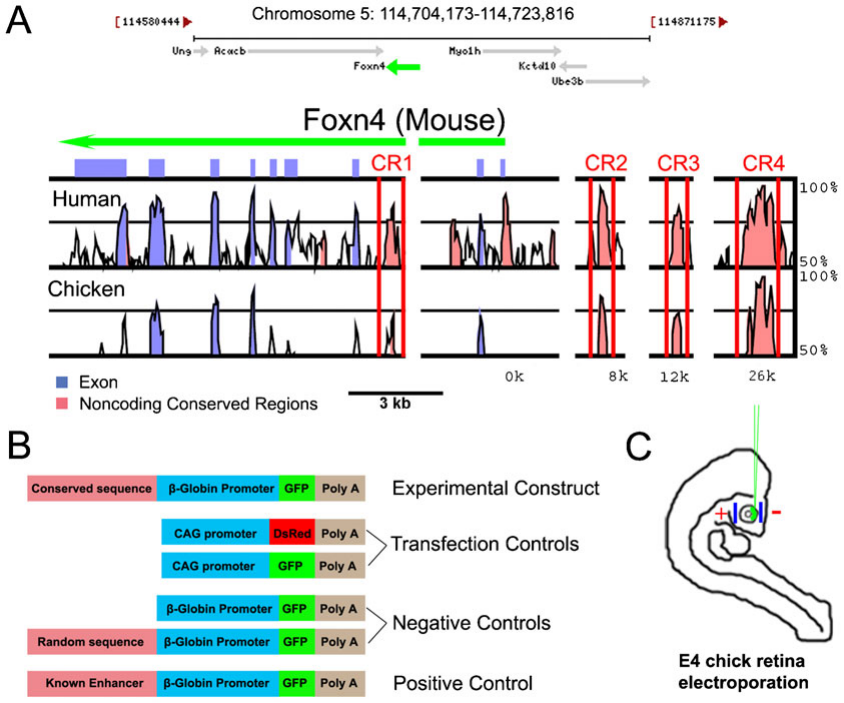
Prediction of Foxn4 *cis*-regulatory elements and experimental design for functional verification. (A) Comparative sequence analysis between mouse and 9 other vertebrate *Foxn4* loci revealed 4 evolutionarily conserved regions (CR). For simplicity, only human, mouse, and chicken alignment is shown here. Blue peaks represent Foxn4 exons while pink peaks represent conserved non-coding sequence. (B) Design of plasmid reporter constructs for experimental construct, and various control constructs, i.e. the negative control, transfection control, and positive control. The experimental construct contains an enhancer candidate upstream of a minimal β-globin promoter and a reporter GFP. Negative control constructs contain the minimal β-globin promoter and the reporter GFP without an inserted sequence or with a random sequence of comparable size. The transfection control contains a strong ubiquitous CAG promoter (chicken β-actin promoter with CMV enhancer), which is in place of the β-globin minimal promoter. The positive control contains a known enhancer, e.g. the RER enhancer (Nie et al., 1996) for photoreceptors, to ensure GFP is expressed in a cell-type specific manner in the presence of a functional enhancer and the β-globin minimal promoter. (C) A mixture of plasmid DNA constructs including the experimental constructs and transfection control, CAG-DsRed was injected and electroporated into the chick retina at embryonic day 4 (E4) to transfect the retinal progenitor cells.

Mouse *Foxn4* gene spans 19 kb and is bracketed by two intergenic regions: 83 kb upstream of *Myo1h* and 4 kb downstream of *Acacb*. To gain insight into the regulation of *Foxn4* expression, we performed comparative DNA sequence analysis to identify evolutionarily conserved noncoding sequences that may serve as *cis*-elements. The intergenic sequences spanning the 5′ and 3′ regions of *Foxn4* from various species, including human, mouse, chicken and other vertebrate species were aligned using multi-LAGAN/mVISTA (Brudno et al., 2003; Frazer et al., 2004) (Fig. 1A; supplementary material Fig. S1). The resulting alignment revealed four highly conserved regions, and thus, predicted them as potential *cis*-elements for Foxn4 (CR1–CR4, pink peaks between red bars in Fig. 1A). CR1 resides within the intronic region of the Foxn4 gene, while CR2–CR4 are located upstream of Foxn4.

### CR1 and CR4 possess gene regulatory activity in the developing retina of both chick and mouse

**Fig. 2.**
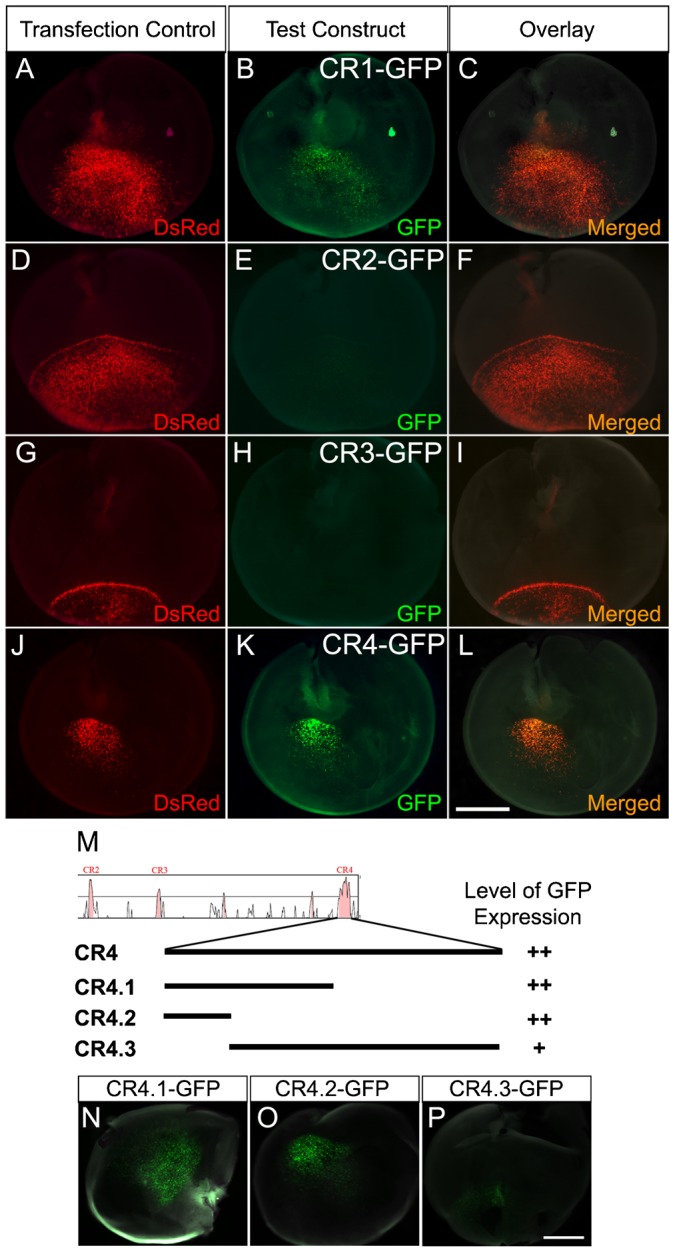
Conserved regions of Foxn4 direct GFP expression in the embryonic chick retina. Chick retinas were injected and electroporated with a mixture of CAG-DsRed (transfection control) and one of the four experimental constructs containing CR1–CR4 on embryonic day 4 (E4). Transfected retinas were harvested at E6. Dissected whole-mount retinas were examined for reporter GFP expression. Successful electroporation was confirmed by DsRed expression (A,D,G,J). Samples resulted from transfection of CR1-GFP (B) and CR4-GFP (K) were shown to contain GFP+ cells (green). However, no GFP expression was observed from CR2 (E) and CR3 (H). (M) Schematic of three highly conserved sub-regions in CR4 (CR4.1–4.3). These three subregions were tested for their ability to direct GFP expression. (N–P) E6 chick retinas two days after electroporated at E4. Similar to CR4, GFP expression can be seen with CR4.1 and CR4.2, and weak GFP expression with CR4.3. Scale bars: 1 mm.

To determine whether the evolutionarily conserved DNA elements (CR1–CR4) have the ability to direct gene expression in retinal development, each of the four conserved regions (Fig. 1B) was individually tested in the developing retina of both chick and mouse using *in ovo* (Doh et al., 2010; Islam et al., 2012) and *ex vivo* (Petros et al., 2009) electroporation methods, respectively. A mixture of DNA constructs including an experimental construct and a transfection control (pCAG-DsRed) was injected and electroporated into the chick retina at embryonic day 4 (E4) or mouse retina at E15 to transfect the retinal progenitors (Fig. 1C). Reporter GFP expression was detected with two constructs (i.e. Foxn4CR1-βGP-GFP (CR1-GFP) and CR4-GFP) in the retina of both the chick (Fig. 2) and mouse (supplementary material Fig. S2).

For negative controls, βGP-GFP or βGP-GFP with a random sequence (Fig. 1B) failed to direct reporter GFP expression (data not shown). As a positive control, βGP with the known enhancer RER for the Rhodopsin gene (Nie et al., 1996), was able to direct photoreceptor-specific GFP expression confirming the ability of the reporter construct to direct cell-specific reporter expression (supplementary material Fig. S3). These results indicate that *cis*-elements CR1 and CR4 possess gene regulatory activity during early retinal development. Since CR4 showed a stronger activity and is the highest conserved *cis*-element, its gene regulatory activity was further analyzed in this study.

### Gene regulatory activity exists in a 129 bp DNA fragment of CR4

To determine the minimum functional DNA element, three highly conserved subregions of CR4 (Fig. 2M) were individually tested for their ability to direct GFP expression. We found two distinct subregions (CR4.2 and CR4.3) possess the ability to direct reporter GFP expression in chick retinas (Fig. 2N–P). However, the level of GFP expression driven by CR4.2 was higher compared to CR4.3. CR4.2 contains the first 129 bp in CR4. Sequence alignment analysis revealed 2 highly conserved motifs across phylogeny among 11 related vertebrate species in CR4.2 (supplementary material Fig. S4A,B). Therefore, CR4.2 was determined as a minimum functional *cis*-element.

### CR4.2 is preferentially active in Foxn4-expressing cells in the developing chick retina

The spatiotemporal gene regulatory activity of CR4.2 in the developing chick retina was further examined. CR4.2-GFP expression is detectable as early as E4.5–E5 in the developing chick retina about 12 hours after electroporation (supplementary material Fig. S5). The highest level of GFP expression was detected at E6 and E7 (supplementary material Fig. S5F,I), very weak expression was observed at E8 (supplementary material Fig. S5K,L), and no GFP expression after E9 (data not shown). This temporal CR4.2-GFP expression pattern (supplementary material Fig. S5C,F,I,L) is similar to CR4-GFP expression (supplementary material Fig. S5B,E,H,K), and consistent with the endogenous Foxn4 expression during retina development in chick (Boije et al., 2008).

**Fig. 3.**
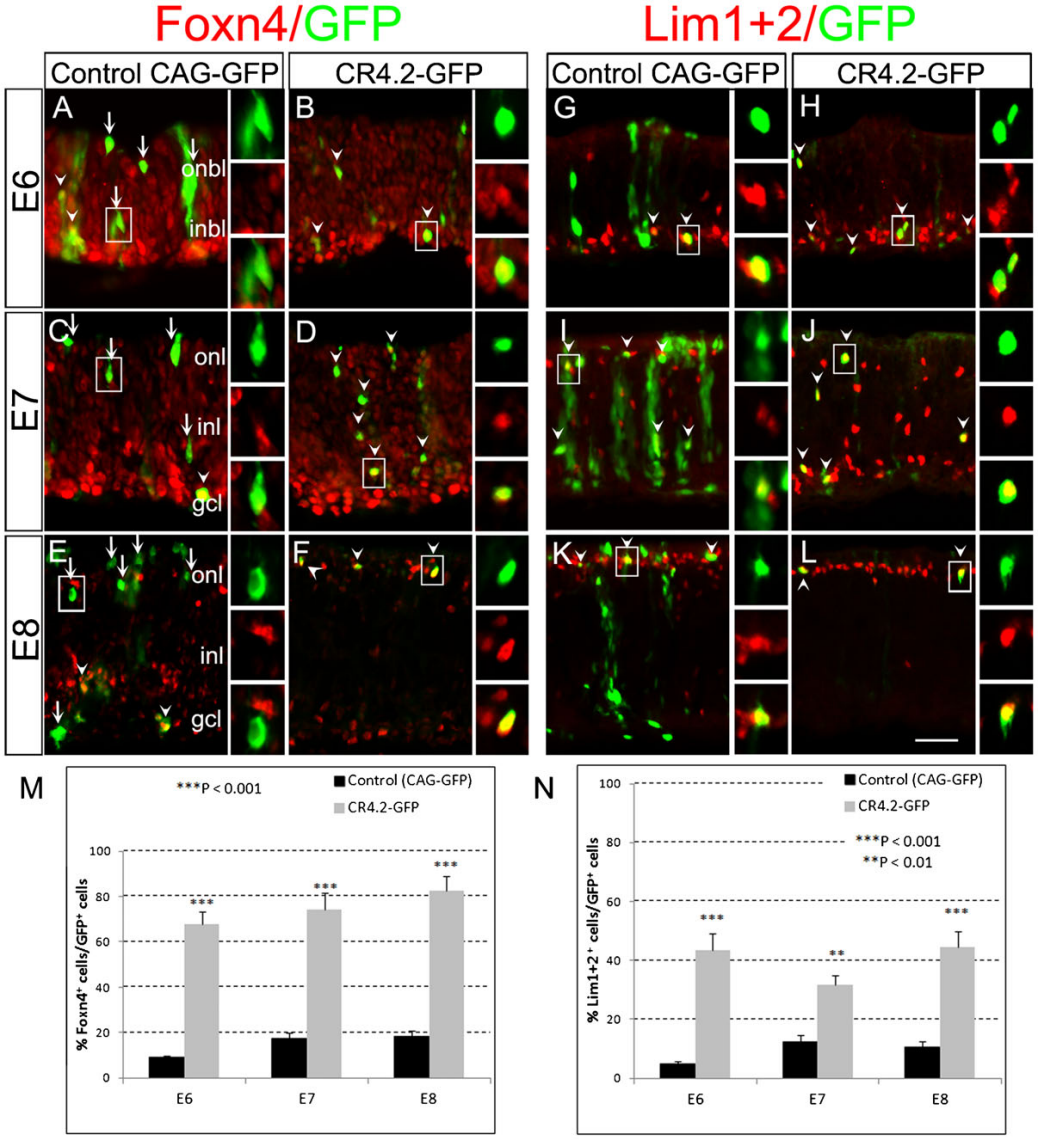
CR4.2 directs GFP expression in Foxn4^+^ cells and differentiating horizontal cells. Chick retinas were electroporated with either the control CAG-GFP construct or CR4.2-βGP-GFP (CR4.2-GFP) construct at embryonic day 4 (E4). Transfected retinas were harvested at E6, E7, and E8 during development, sectioned, and immunostained for GFP (green), Foxn4 (red, panels A–F), and Lim1+2 (red, panels G–L). (A–F) Double labeled cells (boxed regions) are shown in higher magnification on the right (indicated by arrowheads; arrows point to Foxn4-negative cells). (M,N) Quantification of double labeled cells (GFP^+^/Foxn4^+^ or GFP^+^/Lim1+2^+^). Error bars represent standard error of the mean. Data represent the mean ± s.d.; *n*≥3. ONBL, outer neuroblastic layer; INBL, inner neuroblastic layer; ONL, outer nuclear layer; INL, inner nuclear layer; GCL, ganglion cell layer. Scale bar: 20 µm.

To determine whether CR4.2 activity accurately recapitulated some or all of the Foxn4 expression in the retinal cells, CR4.2-GFP expression pattern was compared with the endogenous Foxn4 expression and contrasted with the control CAG-GFP expression at a cellular level (Fig. 3). In contrast to the control CAG-GFP^+^ cells (Fig. 3A,C,E), a significantly higher percentage of CR4.2-GFP^+^ cells were co-stained with Foxn4 at all three stages (67.6% at E6; 74.2% at E7; 82.7% at E8; *n* = 3) (Fig. 3B,D,F,M). At E8 when horizontal and amacrine cells were more mature, we observed that CR4.2-GFP^+^/Foxn4^+^ cells were in a distinct laminar location where the horizontal and amacrine cells reside (arrowheads in Fig. 3F). In contrast, the percentages of the control CAG-GFP^+^ cells co-stained with Foxn4 were about 9.0% at E6, 12.2% at E7, and 18.3% at E8 (Fig. 3M). The increasing percentage of co-stained cells in both the experimental and control groups is well correlate with the differentiation and maturation process of the horizontal and amacrine cells in the retina. This result indicates that CR4.2 activity preferentially occurs in the Foxn4-expressing cells.

### CR4.2 directs GFP expression primarily in horizontal cells

**Fig. 4.**
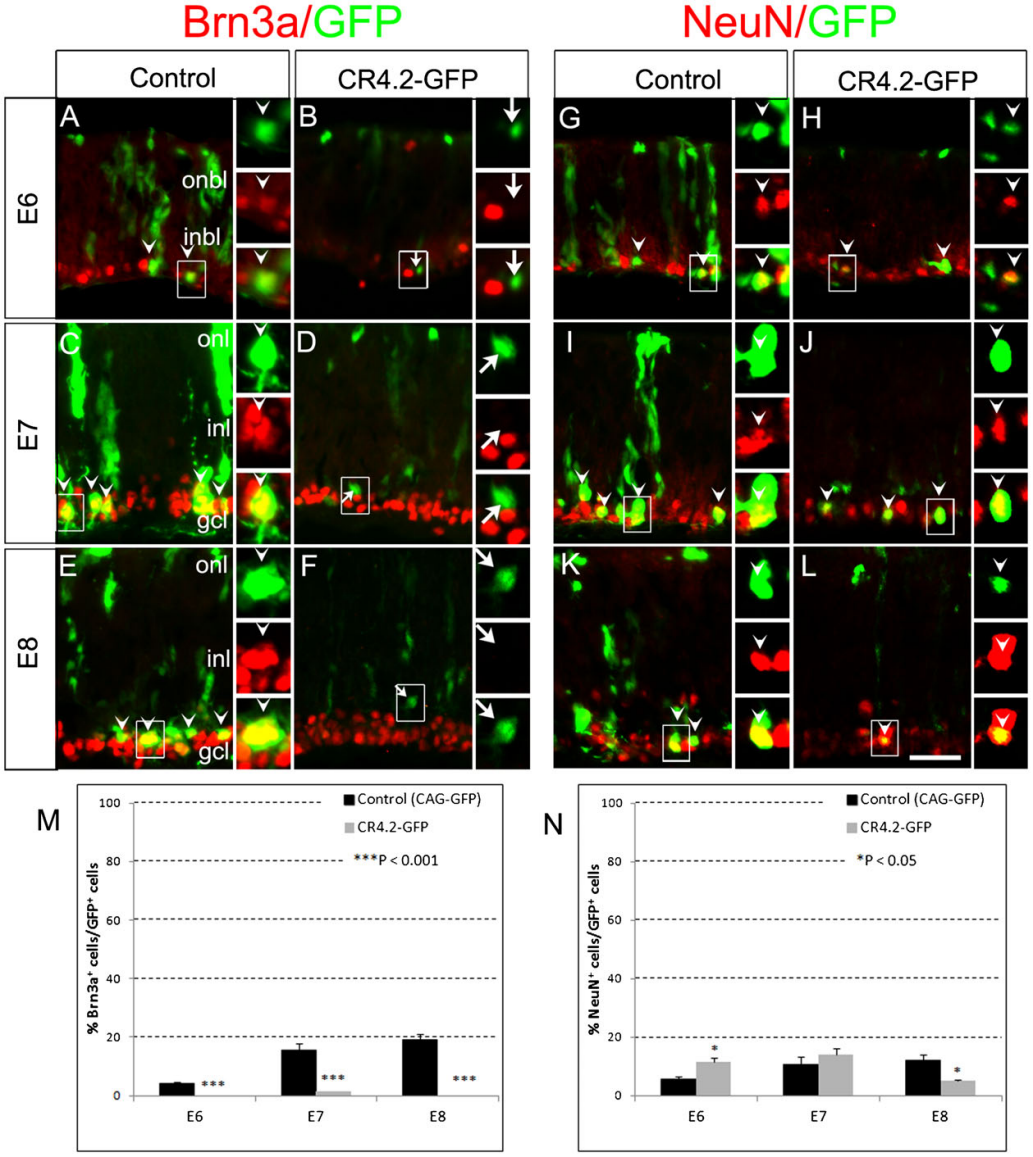
CR4.2 may be active in amacrine cells but not in ganglion cells. Chick retinas were electroporated with either the control CAG-GFP construct or CR4.2-βGP-GFP (CR4.2-GFP) construct at embryonic day 4 (E4). Transfected retinas were harvested at E6, E7, and E8, sectioned, and immunostained for GFP (green), Brn3a (red, panels A–F), and NeuN (red, panels G–L). (A–F) Double labeled cells (boxed regions) are shown in higher magnification on the right (indicated by arrowheads; arrows point to Brn3a-negative cells). (M,N) Quantification of double labeled cells (GFP^+^/Brn3a^+^ or GFP^+^/NeuN^+^). Error bars represent standard error of the mean. Data represent the mean ± s.d.; *n*≥3. ONBL, outer neuroblastic layer; INBL, inner neuroblastic layer; ONL, outer nuclear layer; INL, inner nuclear layer; GCL, ganglion cell layer. Scale bar: 20 µm.

To determine the cell-specific activity of CR4.2, transfected retinal sections at E6, E7 and E8 after electroporation at E4 were stained with cell type-specific markers, e.g. Lim1+2 for horizontal cells (Edqvist et al., 2006; Poché et al., 2007; Boije et al., 2008; Margeta, 2008; Suga et al., 2009), Brn3a for ganglion cells (Liu et al., 2000; Huang et al., 2001; Badea et al., 2009; Nadal-Nicolás et al., 2009), NeuN for ganglion and amacrine cells (Mullen et al., 1992; Doh et al., 2010), and Visinin for cone photoreceptors (Yamagata et al., 1990). We found that the percentage of Lim1+2^+^ cells among CR4.2-GFP^+^ cells (i.e. 43.3% at E6; 31.5% at E7; and 44.5% at E8; *n* = 3) was dramatically higher than that of among the control CAG-GFP^+^ cells (4.9% at E6; 12.1% at E7; and 10.5% at E8; *n* = 3) (Fig. 3G–L,N). The percentage of NeuN^+^ cells among CR4.2-GFP^+^ cells was significantly higher than that among CAG-GFP^+^ cells at E6, but no detectable difference at E7, and lower at E8 (Fig. 4G–L,N). Interestingly, only a few of CR4.2-GFP^+^ cells were co-stained Brn3a, which was dramatically lower than that of CAG-GFP^+^ cells (Fig. 4A–F,M). Since almost none of the CR4.2-GFP^+^ cells were co-labeled with Brn3a (Fig. 4M), the CR4.2-GFP^+^/NeuN^+^ cells were most likely amacrine cells. In addition, among CR4.2-GFP^+^ cells, the percentage of Visinin^+^ cells was dramatically lower than those among CAG-GFP^+^ cells (supplementary material Fig. S6). These results suggest that CR4.2 activity preferentially occurs in horizontal and amacrine cells and may not be in ganglion or cone photoreceptor cells.

### Specific nuclear factors bind to CR4.2

The ability of CR4.2 to direct cell-specific reporter GFP expression is associated with its *trans*-acting protein factors. To identify the binding factors that may interact with CR4.2, we first used MatInspector in Genomatix Suite (München, Germany) (Quandt et al., 1995; Werner, 2000; Cartharius et al., 2005) to search for potential *trans*-acting factor binding sites on CR4.2. The search resulted in 29 potential factor binding sites (supplementary material Fig. S4C).

**Fig. 5.**
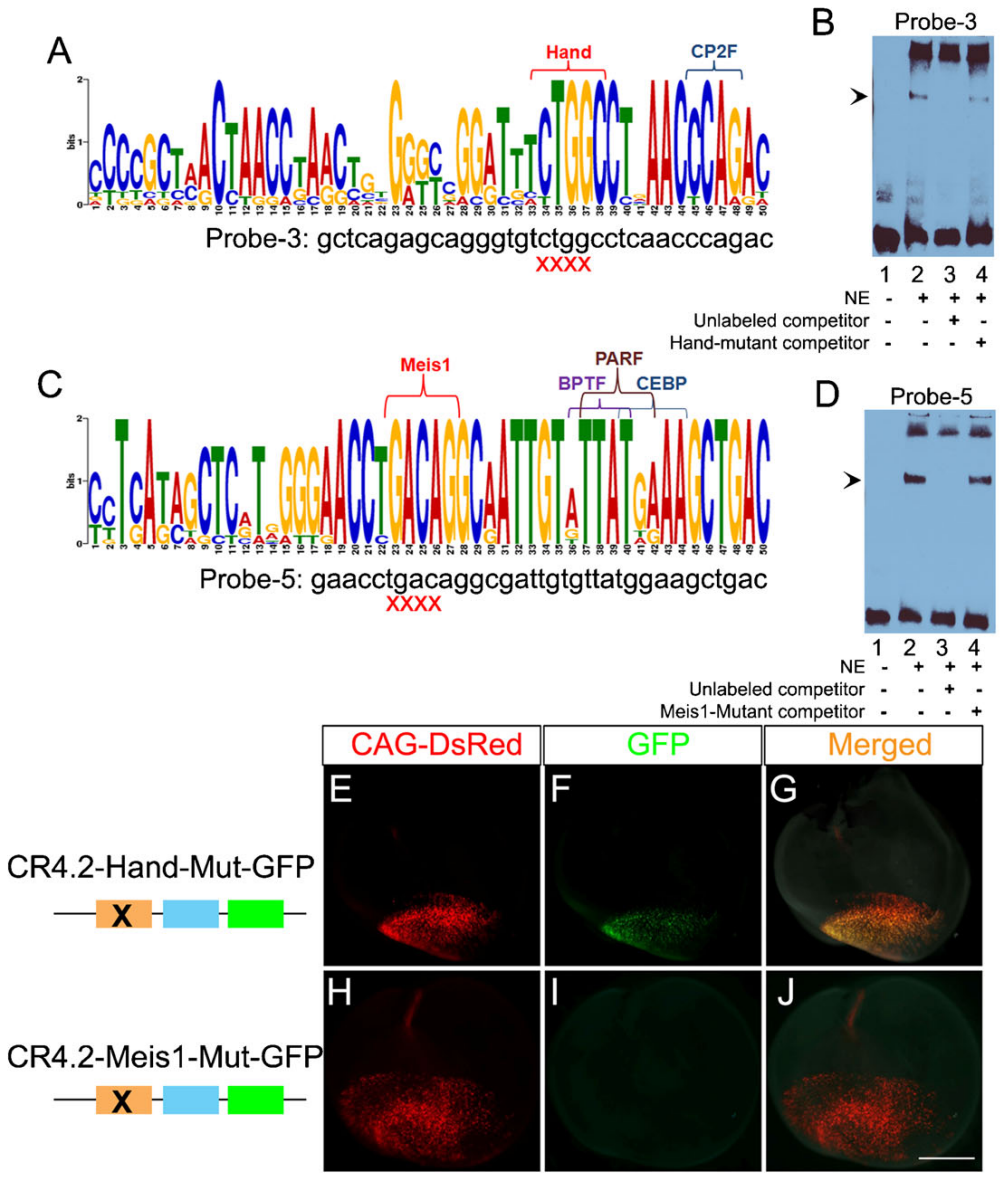
Meis1 transcription factor is essential for CR4.2-GFP expression. Analysis of homologous CR4.2 sequences from 11 species by Motif-based sequence analysis tools MEME (Bailey et al., 2006) revealed two highly conserved motifs (A,C). Electrophoretic mobility shift assays (EMSA) were performed using nuclear extracts isolated from E6 chick retina. Probe-3 (lane 2 in panel B) and Probe-5 (lane 2 in panel D) resulted in a band shift (arrowhead). This band disappeared with unlabeled competitors (lane 3); and the band reappeared with mutant competitors (lane 4). Mutant probes were synthesized with a 4 bp deletion of the TFBSs Hand (CTGG) (A) and Meis1 (TGAC) (C). (E–J) Chick retinas were injected and electroporated with a mixture of control CAG-DsRed (E,H) and a CR4.2-GFP mutant constructs (F,I) on embryonic day 4 (E4). Mutant constructs were generated by site directed mutagenesis. Transfected retinas were harvested and examined for GFP expression at E6. No change in reporter GFP expression was observed with deletion of Hand site (F); while no GFP expression was detected with deletion of Meis1 site (I). Scale bar: 1 mm.

Electrophoretic mobility shift assay (EMSA) was then performed to determine CR4.2 sequence-specific binding with *trans*-acting factors using five short double stranded DNA probes (<40 bp) designed to cover the whole 129 bp (green arrows in supplementary material Fig. S4C). Probe-3 and Probe-5 showed sequence-specific binding activity (Fig. 5; Table 2). Interestingly, both Probe-3 and Probe-5 reside within the two highly conserved motifs (Fig. 5A,C; supplementary material Fig. S4A,B). Probe-3 contains predicted binding sites for transcription factors HAND (Heart- and Neural crest Derivatives) and CP2F (CP2-erythrocyte Factor); and Probe-5 contains predicted binding sites for Meis1 (Myeloid Ecotropic viral Integration Site1), BPTF (Bromodomain and PHD domain transcription factors), PARF (PAR/bZIP family), and CEBP (Ccaat/Enhancer Binding Protein).

To determine which specific factors bind to CR4.2, Probe-3 and Probe-5 were mutated by deleting a 4 bp core motif at the predicted binding sites (Fig. 5A,C; Table 2). EMSA results showed that the factors Hand and Meis1 may bind with CR4.2 (Fig. 5B,D). None of the other tested sites (i.e. CP2F, BPTF, CEBP, and PARF) showed binding activity with CR4.2 (supplementary material Fig. S7).

### Meis1 is necessary for CR4.2-GFP expression

The importance of Hand and Meis1 in regulating CR4.2-GFP expression was then tested using *in ovo* electroporation reporter assay. Mutant reporter constructs, CR4.2-mut-Hand-βGP-GFP and CR4.2-mut-Meis1-βGP-GFP, were generated using site-directed mutagenesis method by deleting a 4 bp core binding motif of Hand and Meis1, respectively (Fig. 5). Chick retinas electroporated with CR4.2-Hand-mutant construct showed no change in GFP expression as compared to CR4.2-GFP expression (Fig. 5E–G), while transfection of CR4.2-mut-Meis1-βGP-GFP construct diminished GFP expression (Fig. 5H–J). This indicates that the binding site of Meis1 (not Hand) is essential for the gene regulatory activity of CR4.2.

### Meis1 is expressed in CR4.2-GFP^+^ and Foxn4^+^ cells

**Fig. 6.**
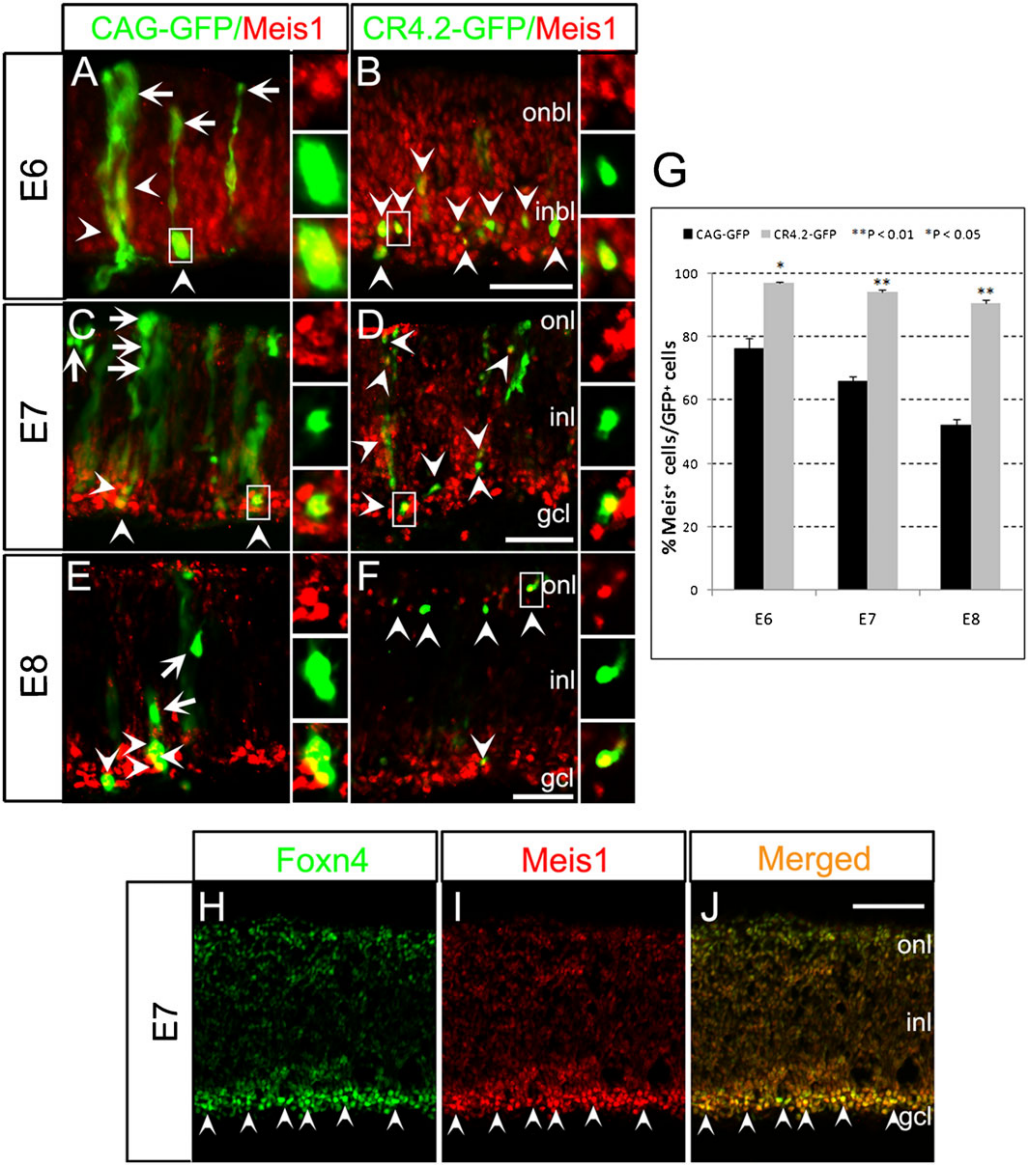
Meis1 protein is present in CR4.2-GFP^+^ and Foxn4^+^ cells. Chick retinas were electroporated with either the control CAG-GFP construct or CR4.2-GFP construct at E4. Transfected retinas were harvested at E6(A-B), E7(C-D), E8(E-F), sectioned, and immunostained for GFP (green) and Meis1 (red). (G) Quantification showed that a significantly high percentage of CR4.2GFP^+^ cells were co-labeled with Meis1as compared with the control CAG-GFP^+^ cellsat E6, E7 and E8 arrowheads in B, D, and F). Error bars represent standard error of the mean. Each histogram represents the mean ± s.d.; *n*≥3. (H-J) At E7, the majority of Foxn4^+^ cells were co-labeled with Meis1 staining (arrowheads). ONBL, outer neuroblastic layer; INBL, inner neuroblastic layer; ONL, outer nuclear layer; INL, inner nuclear layer; GCL, ganglion cell layer. Scale bars = 20 µm.

Since the Meis1 binding site is necessary for CR4.2-GFP expression, we confirmed the expression of Meis1 protein in CR4.2-GFP^+^ cells using immunohistochemistry (Fig. 6). Although the antibody recognizes both Meis1 and Meis2 proteins, Meis2 expression diminished after E3 in chick retina (Heine et al., 2008). Thus, the antibody should only detect Meis1 protein. The percentage of Meis1^+^ cells among CR4.2-GFP^+^ cells (96.9% at E6, 94.2% at E7, and 90.6% at E8; *n* = 3) was significantly higher than that among the control CAG-GFP^+^ cells (76.3%, 66.1%, and 52.2%, respectively) (Fig. 6A–G).

Next, double immunostaining was performed to co-label retinal cells with Meis1 and Foxn4. The staining revealed that almost all of Foxn4^+^ cells were co-labeled with Meis1 in E7 chick retina (Fig. 6H–J). Thus, these results support a role for Meis1 in the regulation of CR4.2-GFP expression and Foxn4^+^ cell development.

### Knockdown of Meis1 abolishes CR4.2-GFP expression

**Fig. 7.**
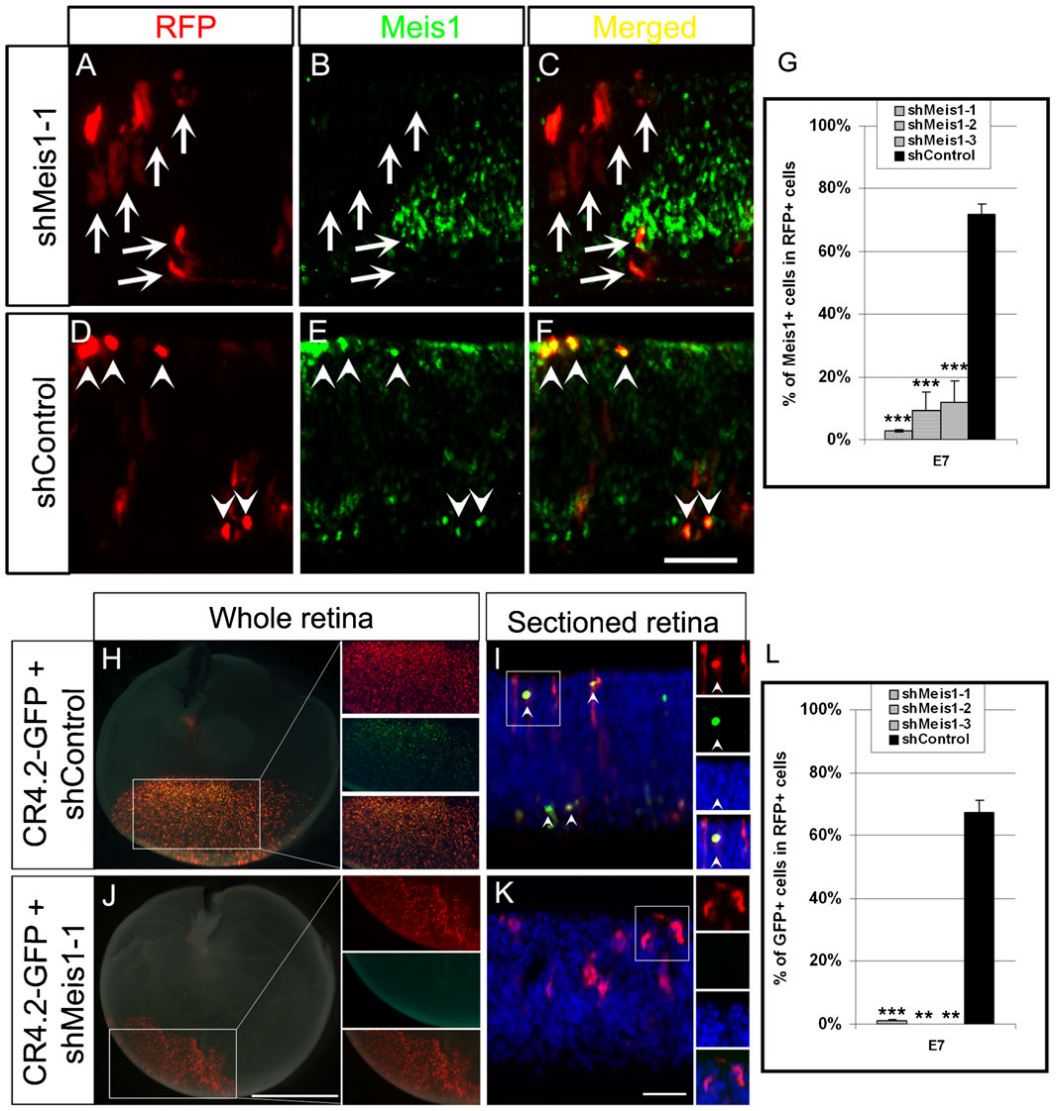
Knockdown of Meis1 abolishes CR4.2-GFP expression. (A–F) Chick retinas were electroporated with Meis1-1-shRNA-RFP (shMeis1-1) or Control-shRNA-RFP (shControl) plasmid at E4. Transfected retina tissues were harvested at E7, sectioned, and immunostained with Meis1 (green). RFP^+^ cells generated by shMeis1 transfection were observed with reduced protein level of Meis1 by antibody staining (arrows in panel B), but was unaffected by control shRNA transfection (arrowheads in panel E). A histogram (G) shows that there was a dramatic decrease in the percentage of Meis1^+^/RFP^+^ cells in shMeis1-1 group. Chick retinas were injected and electroporated with a mixture of CR4.2-GFP and either a shControl (H,I) or shMeis1-1 (J,K) on embryonic day 4 (E4). Transfected retinas were examined for reporter GFP expression at E7. GFP^+^ cells were observed in the transfected retinas from shControl (H,I) but not from shMeis1-1 (J,K). Double labeled cells are indicated by arrowheads, while arrows represent cells that are not co-labeled. (L) Quantification of RFP^+^ cells that coexpress GFP. Error bars represent standard error of the mean. Each histogram represents the mean ± s.d.; *n*≥3. ONL, outer nuclear layer; INL, inner nuclear layer; GCL, ganglion cell layer. Scale bars: 20 µm.

We then performed Meis1 knockdown experiments using an RNAi based method to confirm the role of Meis1 in regulating CR4.2 activity. Plasmid vectors containing a short hairpin RNA (shRNA) sequence were designed to specifically target Meis1 and contain red fluorescence protein (RFP) as a reporter. Three different shMeis1-RFP constructs (shMeis1-1, shMeis1-2, and shMeis1-3) were individually electroporated into chick retina at E4. Meis1 expression was examined three days after electroporation at E7 by immunostaining with anti-Meis1 antibody. The percentage of Meis1^+^ cells in RFP^+^ cells is significantly lower in shMeis1 group (2.9% for shMeis1-1; 9.5% for shMeis1-2; 11.9% for shMeis1-3; *n* = 3) than that of the scrambled shRNA-RFP (shControl) (72%) (Fig. 7A–G; supplementary material Fig. S8A).

To test whether knockdown of Meis1 indeed affects CR4.2 activity, chick retinas were co-transfected with shMeis1-RFP and CR4.2-GFP constructs at E4. Results showed that the majority (67% of CR4.2-GFP^+^ cells were co-transfected with the shControl; whereas only a few CR4.2-GFP^+^ cells were observed in shMeis1-RFP^+^ cell population (1% for shMeis1-1; 0% for shMeis1-2 and shMeis1-3) (Fig. 7H–L). This indicates that Meis1 knockdown efficiently abolishes CR4.2-GFP expression.

### Knockdown of Meis1 affects *Foxn4* expression and horizontal cell lineage development

**Fig. 8.**
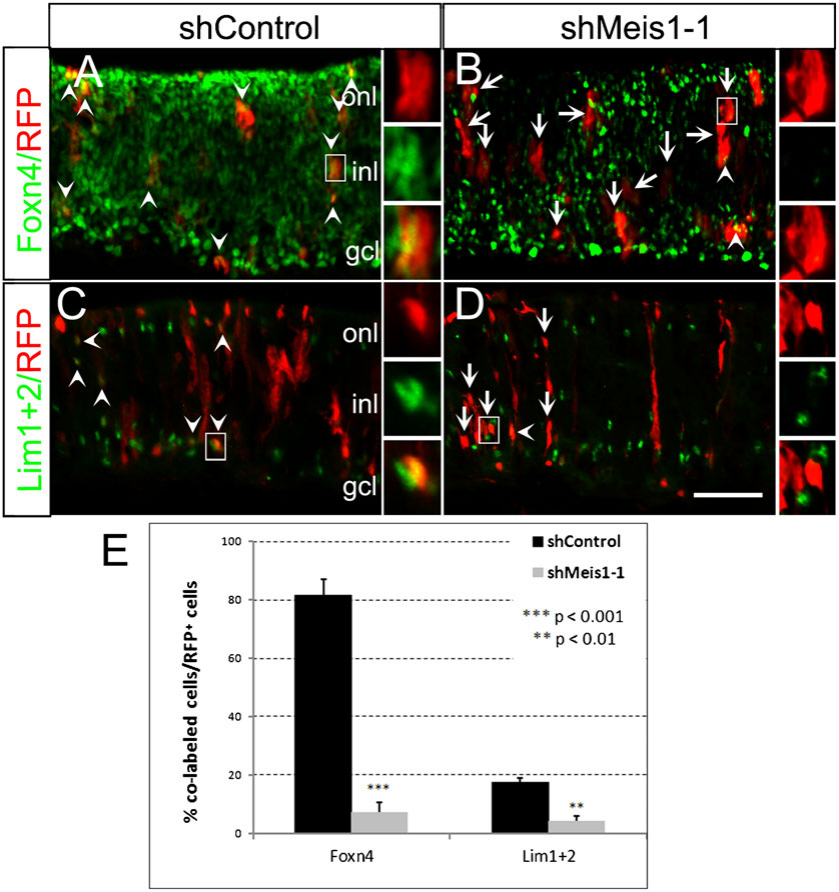
Knockdown of Meis1 reduces the expression of Foxn4 and Lim1+2. Chick retinas were electroporated with shMeis1-1 or shControl construct at E4. Transfected retina tissues were harvested at E7, sectioned, and immunostained for cell specific antibodies: Foxn4 and Lim1+2 (green). RFP^+^ cells show a dramatic reduction of Foxn4 and Lim1+2 expression in the shMeis1-1 (B,D) transfected cells, but not in the shControl transfected cells (A,C). Double labeled cells (Foxn4^+^/RFP^+^ or Lim1+2^+^/RFP^+^) were indicated by arrowheads, while arrows represent RFP^+^ cells that were negative with Foxn4 or Lim1+2 staining. A histogram (E) shows that there was a dramatic reduction of the percentage of RFP+ cells with Foxn4 and Lim 1+2 staining in shMeis1-1 transfected population. Error bars represent standard error of the mean. Each histogram represents the mean ± s.d.; *n*≥3. ONL, outer nuclear layer; INL, inner nuclear layer; GCL, ganglion cell layer. Scale bar: 20 µm.

As shMeis1-RFP transfections decreased Meis1 protein level in RFP^+^ cells and diminished CR4.2-GFP expression (Fig. 7), we next examined whether Meis1 knockdown affects the endogenous level of Foxn4 and horizontal cell lineage development. Transfected cells with Meis1 knockdown in chick retina at E7 three days after electroporation at E4 were immunostained with antibodies against Foxn4 or Lim1+2 (Fig. 8; supplementary material Fig. S8B,C). Compared with the shControl-RFP^+^ cells, there was a significantly lower number of Foxn4^+^ cells (Fig. 8A,B,E) or Lim1+2^+^ (Fig. 8C–E; supplementary material Fig. S8B) in the shMeis1-RFP^+^ cell population (Fig. 8C–E; supplementary material Fig. S8C). Interestingly, the expression of the ganglion cell marker Brn3a and cone photoreceptor marker Visinin was not affected by the Meis1 knockdown (supplementary material Fig. S9). These results suggest that Meis1 transcription factor affects retinal horizontal cell development by regulating *Foxn4* expression via its interaction with *cis*-element CR4.2.

## Discussion

### CR4.2 activity is preferentially in Foxn4+ retinal progenitors and differentiating horizontal cells

We demonstrated that an evolutionarily conserved 129 bp *cis*-element CR4.2 was preferentially active in Foxn4^+^ retinal progenitors, differentiating horizontal cells, and possibly in amacrine cells. CR4.2 activity was observed in E5–E8 chick retina (Fig. 2) and in E15.5–E17.5 mouse retina (supplementary material Fig. S2), a limited developmental time window encompassing cycling retinal progenitors and early postmitotic cells. This activity correlates well with the endogenous Foxn4 expression during retinal development in chick (Boije et al., 2008) and mouse (Gouge et al., 2001; Li et al., 2004). The observation that CR4.2-GFP^+^ cells were co-labeled with Foxn4^+^ cells and Lim1+2^+^ horizontal cells suggests that CR4.2 activity is in the Foxn4^+^ progenitors and differentiating horizontal cells. The fact that CR4.2-GFP^+^ cells were not co-labeled with Brn3a^+^ cells or Visinin^+^ cells (Fig. 4) suggests that CR4.2 activity is not in ganglion cells and cone photoreceptor cells. In fact, retinal ganglion cells and cone photoreceptor cells do not normally express Foxn4 protein (Li et al., 2004). Studies have established that the bipolar neurons and Müller glia were generated in a later developmental stage (Prada et al., 1991; Doh et al., 2010) when Foxn4 expression is lost. It is interesting to notice that NeuN^+^ cells in CR4.2-GFP^+^ cells lower than that of CAG-GFP^+^ cells at E8. Since almost none of the CR4.2-GFP+ cells were co-labeled with Brn3a (Fig. 4M), the CR4.2-GFP+/NeuN+ cells are most likely amacrine cells. Thus, the lower percentage of CR4.2-GFP+/NeuN+ cells at E8 may suggest a decreased CR4.2 activity in amacrine cell lineage development. Together, evidence here supports that CR4.2 is a key *cis*-element that regulates Foxn4 expression in the genesis of the horizontal and amacrine cells.

Although ∼82% of CR4.2-GFP^+^ cells co-labeled with Foxn4 at E8, only ∼44% of CR4.2-GFP^+^ cells were co-labeled with horizontal cell marker Lim1+2 (Fig. 3M,N). This suggests that CR4.2-GFP^+^/Foxn4^+^ includes a population of differentiating horizontal cells and other cell types, e.g. amacrine cells. This is consistent with previous findings that Foxn4^+^ retinal progenitors can give rise to both horizontal and amacrine cells (Li et al., 2004). It is known that heterogeneity exists among horizontal cells in the chick retina (Génis-Gálvez et al., 1981; Tanabe et al., 2006). Thus, CR2-GFP^+^/Lim1+2^+^ cells may only comprise a subpopulation of the horizontal cells. It is also possible that CR4.2 activity might exist in an early phase of the retinal development and our electroporation experiments performed at E4 may capture a fraction of these cells before CR4.2 activity turned off at E8.5.

It is interesting that neither CR2 nor CR3 were able to direct GFP expression in the retina of chick as well as mouse (Fig. 2; supplementary material Fig. S2). This indicates that not all conserved sequences are functional *cis*-elements. However, we cannot rule out the possibility that CR2 and CR3 may function in another development stage, or they are not sufficient to drive gene expression independently.

### CR4.2 activity is regulated by Meis1 transcription factor

Using EMSA, site-directed mutagenesis and shRNA-based gene knockdown assays, we demonstrated that the cell-specific gene regulatory activity of CR4.2 is modulated by Meis1 transcription factor. This is supported by the observation that mutant CR4.2 with Meis1 binding motif deletion failed to direct GFP expression (Fig. 5). In addition, Meis1 knockdown resulted in a significant reduction of Foxn4 expression (Fig. 8A,B,E; supplementary material Fig. S8B) and decreased number of Lim1+2^+^ horizontal cells (Fig. 8C–E; supplementary material Fig. S8C). These data strongly support a critical role for Meis1 in regulating Foxn4 expression and horizontal cell lineage development. Thus, we have not only uncovered a novel role for Meis1 protein in regulating Foxn4 expression but also provided new insights into the molecular mechanism that governs gene regulation in retinal progenitors and cell lineage development.

Previous studies have established that Hox, Pbx and Meis families of transcription factors form heteromeric complexes and bind DNA through specific homeobox domains to regulate gene expression (Ferretti et al., 2006; French et al., 2007; Heine et al., 2008). Thus, it is noteworthy to mention that the Meis1 binding site in CR4.2 is adjacent to the predicted binding sites for Hoxa9 and Pbx1 transcription factors (supplementary material Fig. S4). Hence, it is likely that Meis1 may play a role in Foxn4 expression via its interaction with Hoxa9 and Pbx1. However, additional evidence is needed to confirm this hypothesis.

In summary, we have demonstrated that CR4.2 *cis*-element and its interacting transcription factor Meis1 play important roles in regulating *Foxn4* expression during chick retinal development. These findings provide new insights into molecular mechanisms that govern gene regulation in retinal progenitors and cell lineage development.

## Materials and Methods

### Sequence alignments

Foxn4 sequences from the human, mouse, rat, cow, chicken and other vertebrate genomes were retrieved using NCSRS (Doh et al., 2007) and aligned using multi-LAGAN (Brudno et al., 2003) to identify fragments >100 bp and >75% identity as candidate *cis*-elements. The percent identity and the length of the conserved sequence were used to calculate a score for each conserved region (score = percent identity+(length/60)). Based on this scoring system the percent identity was more heavily weighted to ensure that shorter very highly conserved sequences are not ranked below longer sequences with lower levels of conservation (Fig. 1A).

### DNA plasmids

For testing the regulatory activity of the candidate *cis*-elements, a reporter assay plasmid was designed to contain a *cis*-element, a human minimal basal promoter, β-globin promoter (βGP) (Yee and Rigby, 1993), and a reporter gene, green florescent protein (GFP). Noncoding regions of CR1–CR4 were PCR amplified and inserted in the testing plasmid constructs (Fig. 1B; Table 1). A known enhancer, RER for Rhodopsin gene (Nie et al., 1996), coupled with βGP-GFP was constructed as a positive control. Two plasmid constructs, CAG-GFP or CAG-DsRed (Matsuda and Cepko, 2004), were used as transfection controls (Fig. 1B).

**Table 1.**
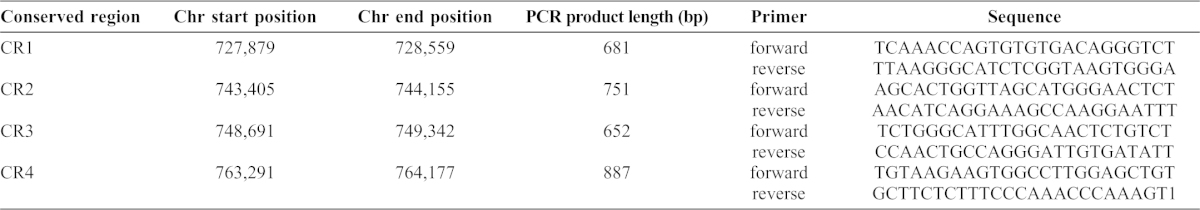
List of evolutionarily conserved regions at *Foxn4* locus and PCR primers for amplifying these regions.

**Table 2.**
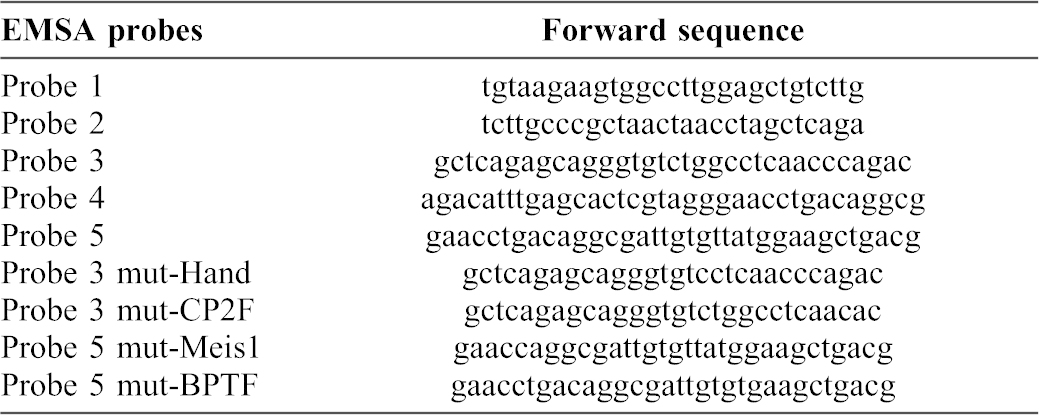
List of probes used in EMSA for CR4.2.

### Chicken and mouse embryos

Fertilized pathogen-free (SPF) white leghorn chicken (*Gallus domesticus*) eggs (Sunrise Farms, Catskill, NY) were incubated at 37.5°C and 60% humidity (GQF manufacturing, Savannah, GA) for 96–100 hours to obtain embryos that are at the developmental stage (Hamburger, 1992; Hamburger and Hamilton, 1992; reprint of 1951 paper) HH22 (∼ embryonic day 4, E4).

Timed-pregnant CD-1 mice were purchased from Charles River Laboratories (Wilmington, MA) and maintained on a 12 hr/12 hr (7:00 A.M. to 7:00 P.M.) light/dark schedule from the time of arrival until the time of the experiment. Pregnancies were timed from the day on which a vaginal plug was detected and designated as embryonic day 0 (E0). All of the animal experiments were approved by the Institutional Animal Care and Facilities Committee at Rutgers University.

### *In ovo* electroporation

Targeted retinal injection and *in ovo* electroporation was performed as described previously (Doh et al., 2010; Islam et al., 2012). Plasmid DNA concentration ranges from 3–6 µg/µl with 0.025% fast green for visualization purpose. Plasmid constructs were directly delivered into the embryonic chick subretinal space (Fig. 1C) and electroporated with 5 square pulses of 15 V for 50 ms with 950 ms intervals using a pulse generator ECM 830 (Harvard Apparatus, Holliston, MA).

### Mouse retinal explant cultures and *ex vivo* electroporation

Mouse retinal explant cultures were prepared as described previously (Tabata et al., 2004). Briefly, retinas derived from mouse embryos were placed on a Millicell chamber filter insert (Millipore). Filters were placed into a six-well plate containing 1 ml of explant medium and cultured. Monolayer culture was set up as described earlier (Matsuda and Cepko, 2004; Koso et al., 2006; Petros et al., 2009). Electroporation was performed using Electroporator BTX ECM 830 (Harvard Apparatus), Round Platinum 2 mm Petridish Electrode, CUY700-P2E and Round Platinum 2 mm Cover Electrode CUY700-P2L (Protech, Boerne, TX).

### Tissue processing and immunohistochemistry

Chick embryos were harvested at three time points (i.e. E6, E7 or E8) after electroporation at E4, and placed in cold PBS, and then fixed in 4% paraformaldehyde (in PBS) for up to 4 hours, and washed in PBS 3 times for 5 minutes at 4°C, and then infiltrated in 30% sucrose in PBS overnight. Retinal tissue sections at 10–15 µm were cut using a cryostat (Thermo 0620E), mounted on Superfrost slides (Fisher Scientific) and air-dried.

Immunostaining was performed using Shandon Slide Rack (Thermo Fisher Scientific, Waltham, MA). Sections were incubated in blocking solution (0.05% Triton X-100, 10% goat serum or donkey serum, 3% BSA in 1× PBS) for 1 hour at room temperature followed by overnight primary antibody application. Primary antibodies and dilutions used were as follows: goat or rabbit anti-GFP (1:500, Abcam), mouse anti-Foxn4 (1:1000, Aviva), mouse anti-Lim1+2 (1:40, 4F2 supernatant, DSHB), mouse anti-Brn3a (1:200, Millipore), mouse anti-NeuN (1:1000, Millipore), mouse anti-Visinin (1:20, 7G4 supernatant, DSHB), and goat anti-Meis1/2 (1:250, Santa Cruz). Slides were then washed with PBS and secondary antibodies carrying fluorescence from the appropriate host were applied (1:300 dilution; Jackson Immuno Research, West Grove, PA). The slides were washed with PBS and cover slipped.

### Imaging

Microscopy and imaging analysis were performed using an upright fluorescence microscope (Zeiss Axio Imager A1) with a monochrome digital camera Axiocam MRM (Zeiss, Germany). Images of GFP-expressing cells and antibody labeled cells (Cy3) were taken separately using 488 nm and 543 nm filters, respectively. Images of Cy3 and GFP channels were then overlaid using Adobe Photoshop CS to create pseudo-colored double-labeled images.

### Data quantification

The minimum number of a particular cell type that was scored ranged from 20 to 140 cells per retina, depending on the abundance within the sample, and each percentage shown in the figures was the combined average for three separate retinas. Error bars in figures represent the standard deviation. In cases where results were tested for statistical significance, a student's t-test was applied with a cutoff of P<0.05.

### Electrophoretic mobility shift assay (EMSA)

Potential transcription factor binding sites were predicted by MatInspector (Genomatix) (Quandt et al., 1995; Werner, 2000; Cartharius et al., 2005). Double stranded DNA probes ranging 30–35 bp were designed to span CR4.2. Probes were synthesized by IDT (Piscataway, NJ) as single stranded oligonucleotides. Single stranded oligonucleotides were biotinylated using Biotin 3′ End DNA Labeling Kit (Thermo Fisher Scientific, Waltham, MA) and annealed at room temperature an hour immediately prior to binding assay. Unlabeled single stranded probes were annealed and used as double stranded competition probes. The ratio of 40:1 was used for competition probe to labeled probes. Nuclear extracts at three different stages were prepared individually from dissected chick retinas at E6, E7 and E8. The EMSA binding reaction and competition reaction were performed according to the LightShift Chemiluminescent EMSA Kit (Thermo Fisher Scientific, Waltham, MA) protocol. The reaction mixture was loaded onto an 8–12% non-denaturing polyacrylamide gel containing 0.5× TBE (40 mM Tris, 40 mM borate, 1 mM EDTA). Mini (8×8×0.1 cm) gels were run at 100 V for 3 h at 4°C and transferred to membrane.

### Site-directed mutagenesis

Mutant constructs were generated using a PCR-based site directed mutagenesis method, as described previously (Nøhr and Kristiansen, 2003). Two sets of mutagenesis primers were designed with a 4 bp deletion for Hand and Meis1 transcription factor binding sites. Mutant constructs were verified by DNA sequencing (Genewiz, Inc., South Plainfield, NJ).

### Meis1 knockdown using shRNA

For RNA interference gene silencing experiment, knockdown of Meis1 expression was performed by transfecting embryonic chick retina with shRNA specific to Meis1 genes or a non-targeting control shRNA (OriGene Technologies, Inc., Rockville, MD). Each shRNA clone was constructed using the pRFP-C-RS vector. The three specific Meis1-targeting sequences were:

shMeis1-1: 5′-AGGTGATGGCTTGGACAACAGTGTAGCT-3′

shMeis1-2: 5′-GTTTGCTCCTCCGAGTCTTTCAATGAAGAC-3′

shMeis1-3: 5′-TGCTCCTCCGAGTCTTTCAATGAAGA-3′

The targeting sequence of shMeis1-1 is based on the conservation between mouse and chicken Meis1 sequence. The targeting sequences of shMeis1-2 and shMeis1-3 designed with chicken Meis1 sequence.

## Supplementary Material

Supplementary Material
