# Supplementary material for: Meis1 regulates Foxn4 expression during retinal progenitor cell differentiation
Source: Biol Open. 2013 Sep 6;2(11):1125–36. doi: 10.1242/bio.20132279 (PMC3828759; doi:10.1242/bio.20132279)
Supplement: Supplementary Material [file supp_2_11_1125__index.html]

Meis1 regulates Foxn4 expression during retinal progenitor cell differentiation — Meis1 regulates Foxn4 expression during retinal progenitor cell differentiation — Supplementary Material 

# Meis1 regulates Foxn4 expression during retinal progenitor cell differentiation

## bio.20132279 Supplementary Material

**Files in this Data Supplement:**

- Supplementary Material - Miriam Luichtl et al. doi: 10.1242/bio.20132279
