# Supplementary material for: Meis1 regulates Foxn4 expression during retinal progenitor cell differentiation
Source: Biol Open. 2013 Sep 6;2(11):1125–36. doi: 10.1242/bio.20132279 (PMC3828759; doi:10.1242/bio.20132279)
Supplement: Supplementary Material [file supp_bio.20132279_bio.20132279-s1.pdf]

Supplementary Material

Mohammed M. Islam et al. **doi: 10.1242/bio.20132279**

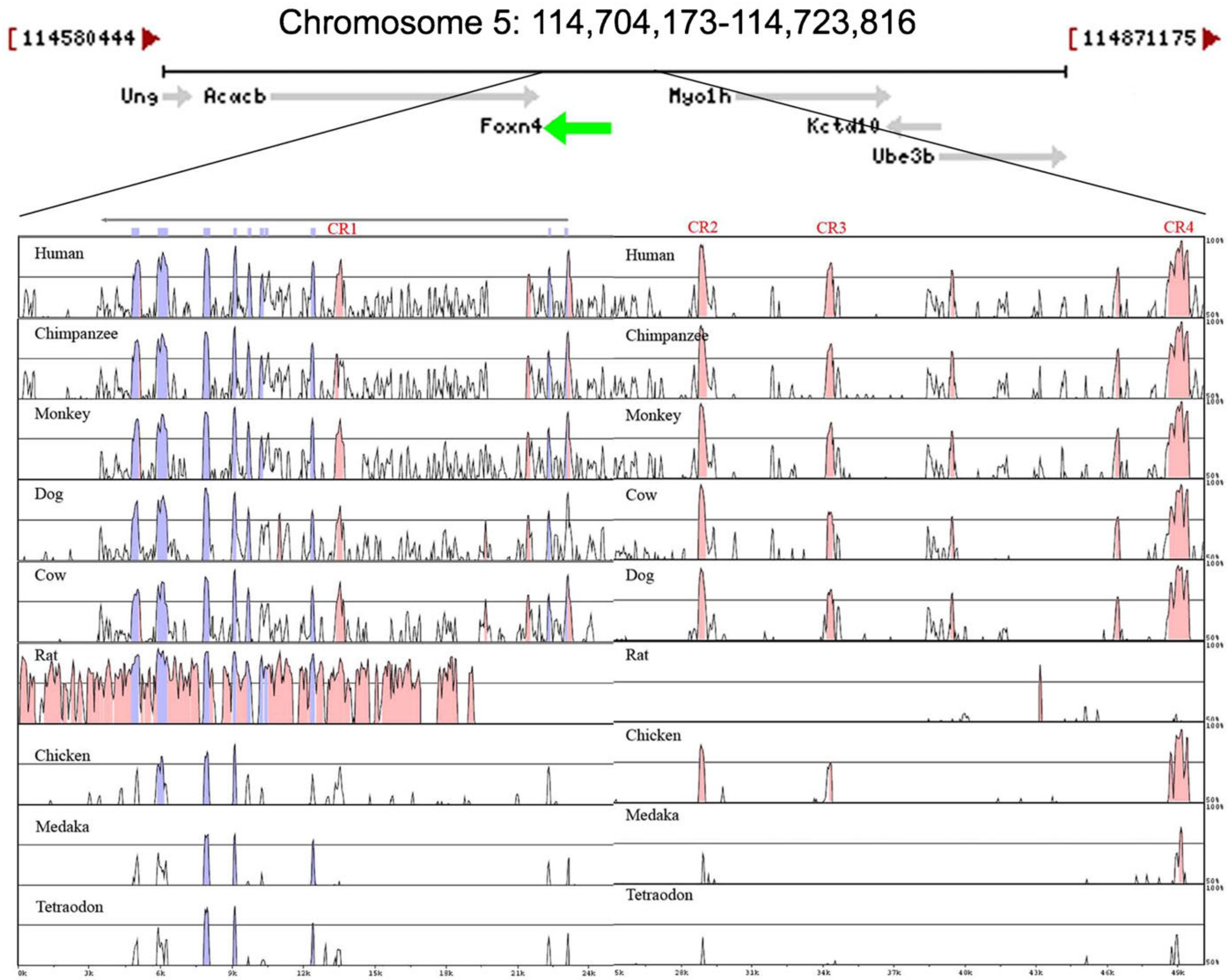

Fig. S1. Computational prediction of highly conserved regions (CR) that surround the mouse Foxn4 gene. The sequences of Foxn4 gene locus from the various genomes including human, cow, dog, rat, opossum, chick, zebrafish, and tetradon were aligned using multi-LAGAN to identify conserved regions (CR) of ≥75% identity over a 100 bp span. The percent identity and the length was used to calculate a score for each CR (score=percent identity+(length/60)). The peaks in the alignment represent the regions with percent identity between 50% and 100%. Pink peaks represent CRs with ≥75% identity. Blue peaks represent Foxn4 exons.

Biology Open

Biology Open

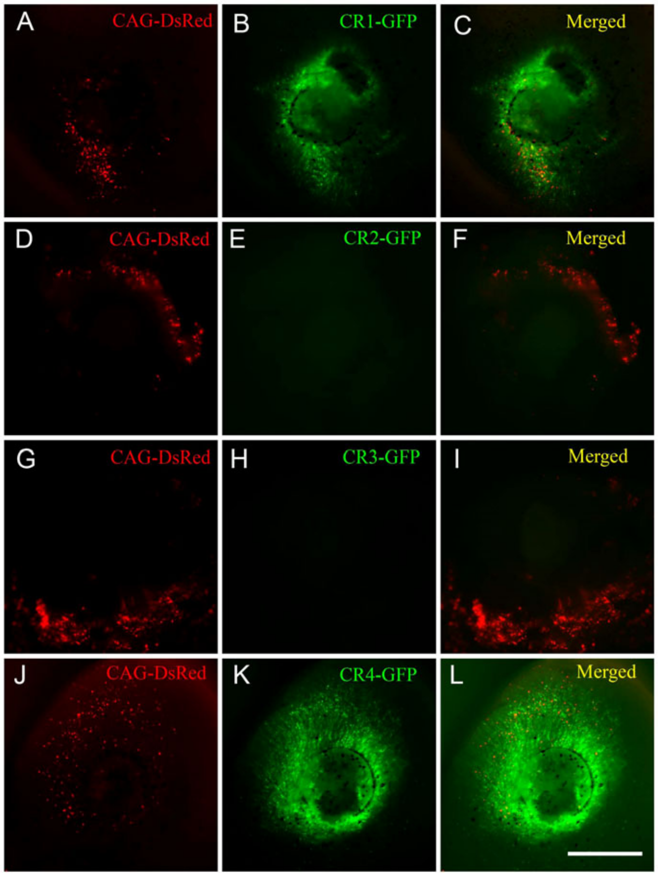

Fig. S2. CR1 and CR4 direct reporter GFP expression in embryonic mouse retina. Mouse retinas were transfected with a mixture of pCAG-DsRed (transfection control) and various enhancer constructs at embryonic day 15 (E15) through *ex vivo* electroporation method. Transfected retinas were examined 65 hours after the electroporation (E 17.5) for reporter gene expression. Successful electroporation was confirmed by DsRed expression (A,D,G,J). Imaging through individual channel shows that CR1 (B) and CR4 (K) has the ability to drive GFP expression. However, CR2 (E) and CR3 (H) do not show any ability to drive gene expression. Scale bar: 500 µm.

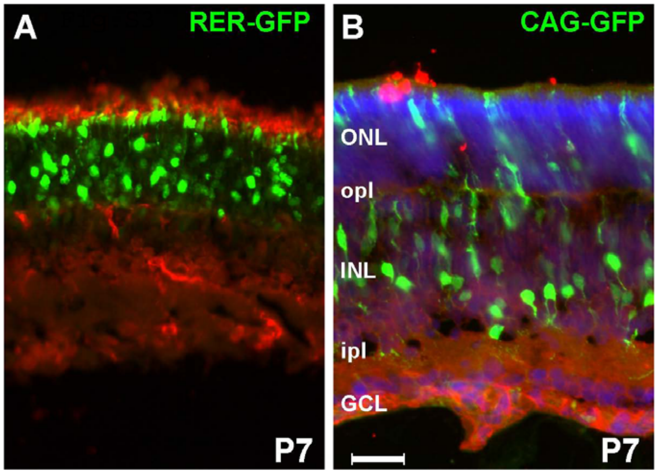

Fig. S3. Photoreceptor specific reporter GFP expression under the control of a known enhancer RER. The reporter GFP expression of postnatal day 7 (P7) developing mouse retina after electroporation of RER-βGP-GFP (RER-GFP) construct (A) and the control CAG-GFP (B) at P0. RER is a previously characterized photoreceptor-specific enhancer (Nie et al., 1996). RER-GFP+ cells were found only in the outer nuclear layer (ONL), where photoreceptors reside; while the control CAG-GFP+ cells were found in both the ONL and the inner nuclear layer (INL). Scale bar: 20 µm.

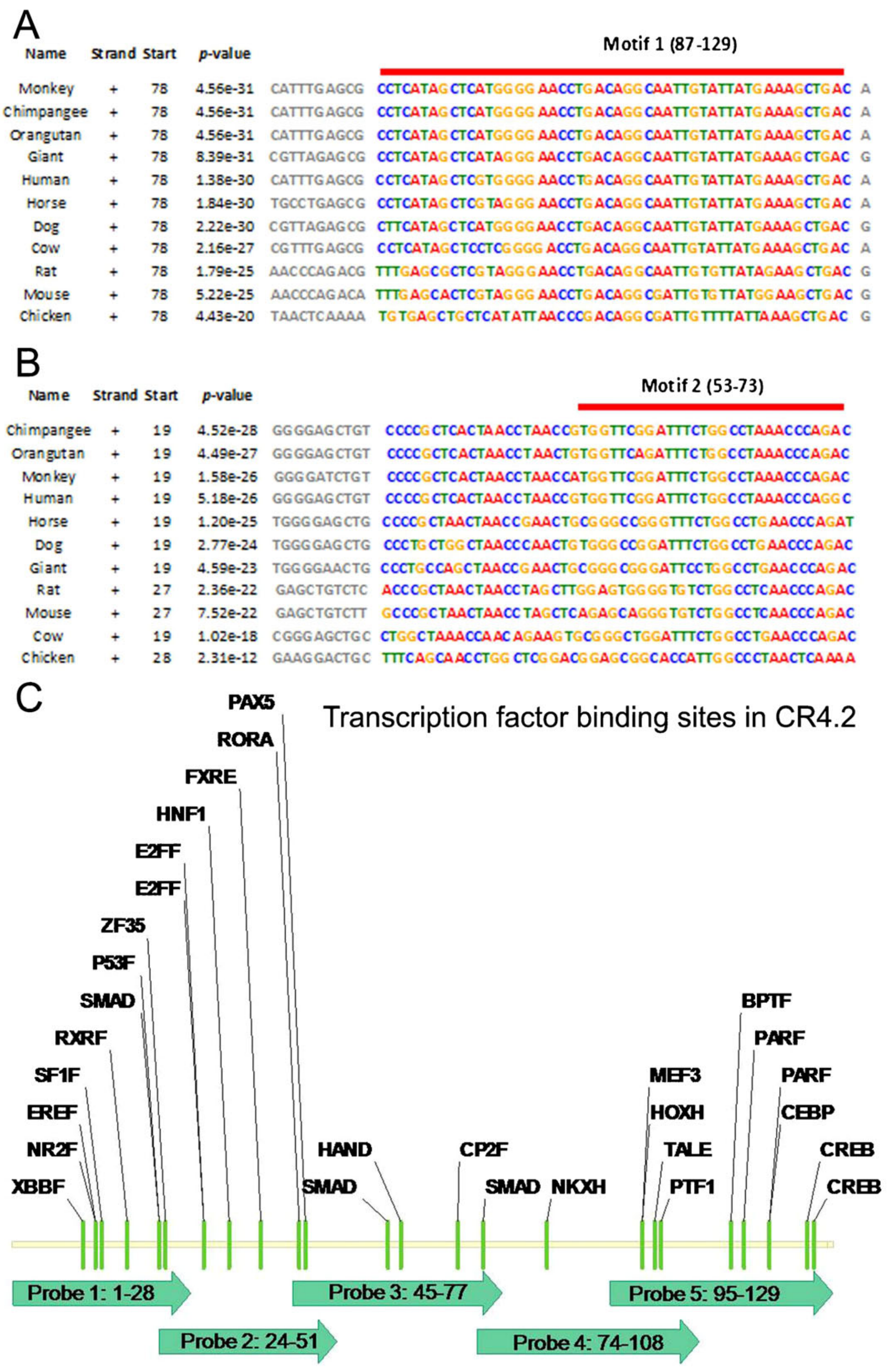

Fig. S4. CR4.2 contains two highly conserved motifs and potential transcription factor binding sites. Sequences of CR4.2 among 11 vertebrate species were aligned and analyzed by MEME (Multiple Em for Motif Elicitation) suite. Each nucleotide is highlighted with a unique color to visualize the conservation among the species. (A,B) Two highly conserved motifs were identified (red lines on top of the alignment).
(C) Using MatInspector in Genomatix, murine CR4.2 sequence was searched for potential transcription factor binding sites. The search resulted in 29 of potential factor binding sites. Green arrows represent 5 overlapping EMSA probes spanning the whole CR4.2 sequence.

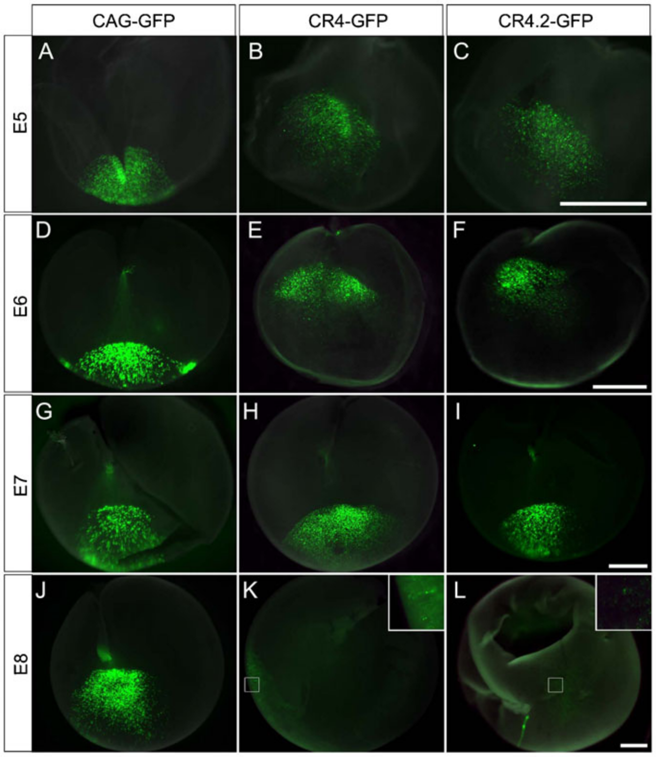

Fig. S5. CR4.2 activity is in the early chick retina. Reporter GFP expression in dissected whole-mount chick retinas at E5 (A–C), E6 (D–F), E7 (G–I), and E8 (J–L) after electroporation with the control CAG-GFP (A,D,G,J), and experimental CR4-GFP (B,E,H,K) and CR4.2-GFP (C,F,I,L) constructs at embryonic day 4 (E4). The reporter GFP expression from the control CAG-GFP was strong and maintained from E5 to E8 (A,D,G,J); while CR4-GFP or CR4.2-GFP expression was diminished by E8 (K,J). The white-boxed region is shown in a higher magnification on the right. Scale bars: 1 mm.

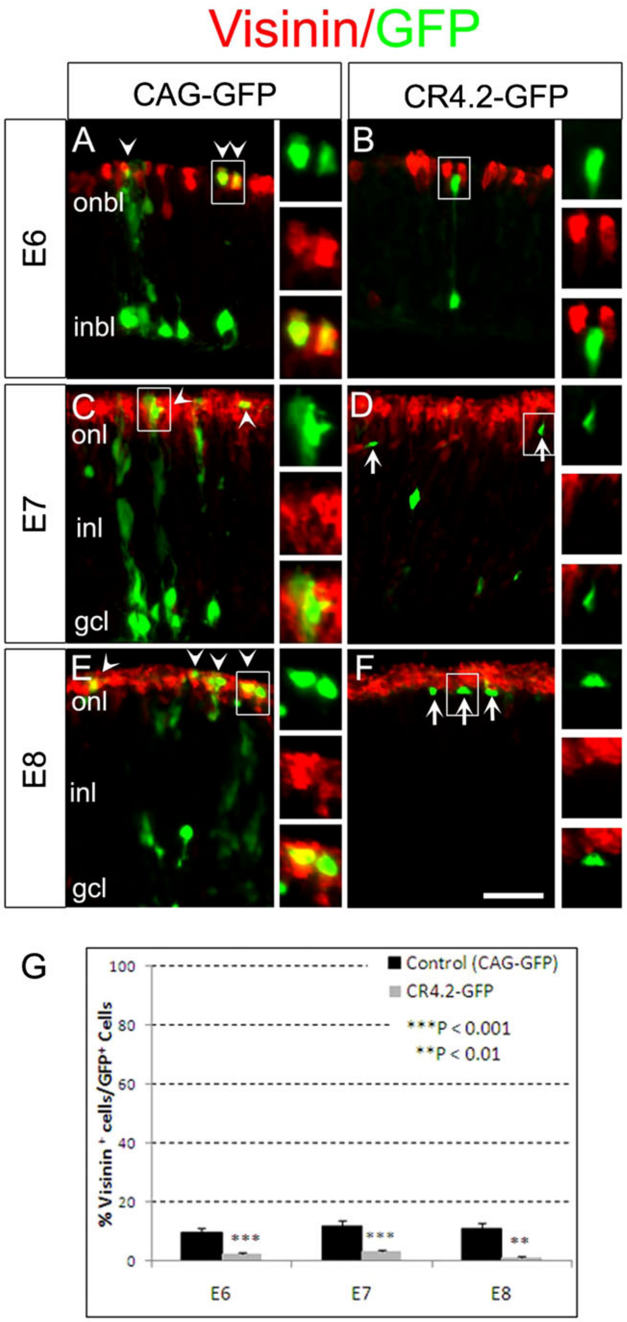

Fig. S6. CR4.2 is not active in photoreceptor cells. Chick retinas were electroporated with either control CAG-GFP construct or CR4.2-βGP-GFP (CR4.2-GFP) construct at embryonic day 4 (E4). Transfected retinas were harvested at E6 (A,B), E7 (C,D), and E8 (E,F), sectioned, and immunostained for GFP (green), Visinin (red). The white-boxed region is shown in higher magnification on the right. Double labeled cells are indicated by arrowheads and arrows point to Visinin-negative cells. (G) Quantification of double labeled cells (GFP+ and Visinin+). The average of three independent experiments is shown; error bars indicate standard deviation. ONBL, outer neuroblastic layer; INBL, inner neuroblastic layer; ONL, outer nuclear layer; INL, inner nuclear layer; GCL, ganglion cell layer. Scale bar: 20 µm.

Biology Open

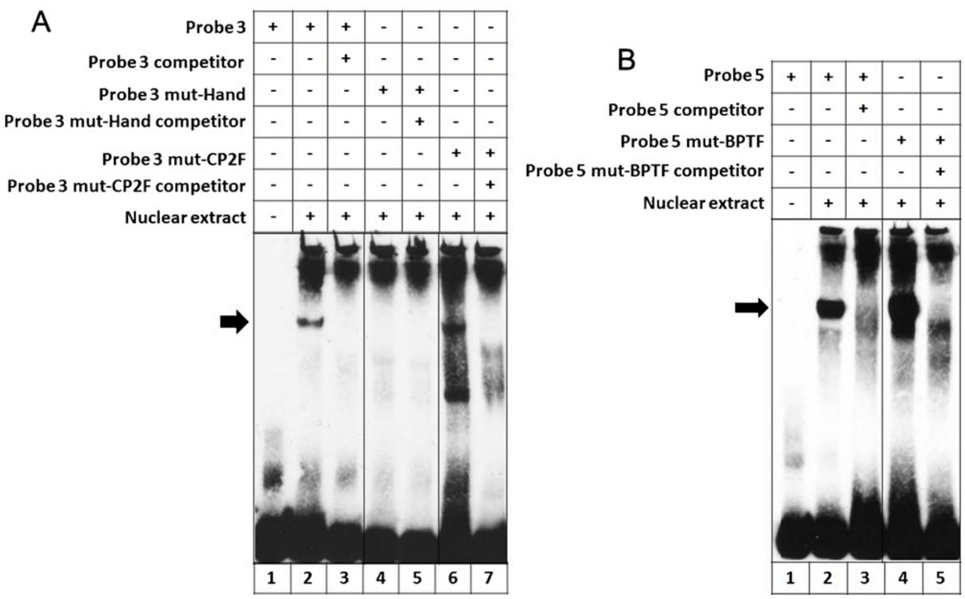

Fig. S7. Identification of transcription factor binding sites in CR4.2. Electrophoretic mobility shift assays (EMSA) were performed using Probe-3, Probe-5 and corresponding mutant probes spanning 45–77 bp and 95–129 bp regions of CR4.2. (A) EMSA gel shows sequence specific binding of Probe-3 as indicated by arrow (lane 2). Binding was competed away using unlabeled competition probe (lane 3). The mutant Hand probe showed no binding (lane 4). Competition with unlabeled mutant Hand probe also showed no binding (lane 5). Mutation in CP2F binding site (Fig. 5) does not affect this binding rather increase binding affinity for other protein (lane 6). Both bands were able to be competed away using the competition probe (lane 7). (B) EMSA gel shows sequence specific binding of Probe-5 as indicated by arrow (lanes 2 and 6). Mutation in BPTF, PARF and CEBP binding site (Fig. 5) does not affect this binding (lane 4). All nuclear extracts used for EMSA were isolated from E6 chick retina. The competition was carried out using 50 fold unlabeled probes.

Biology Open

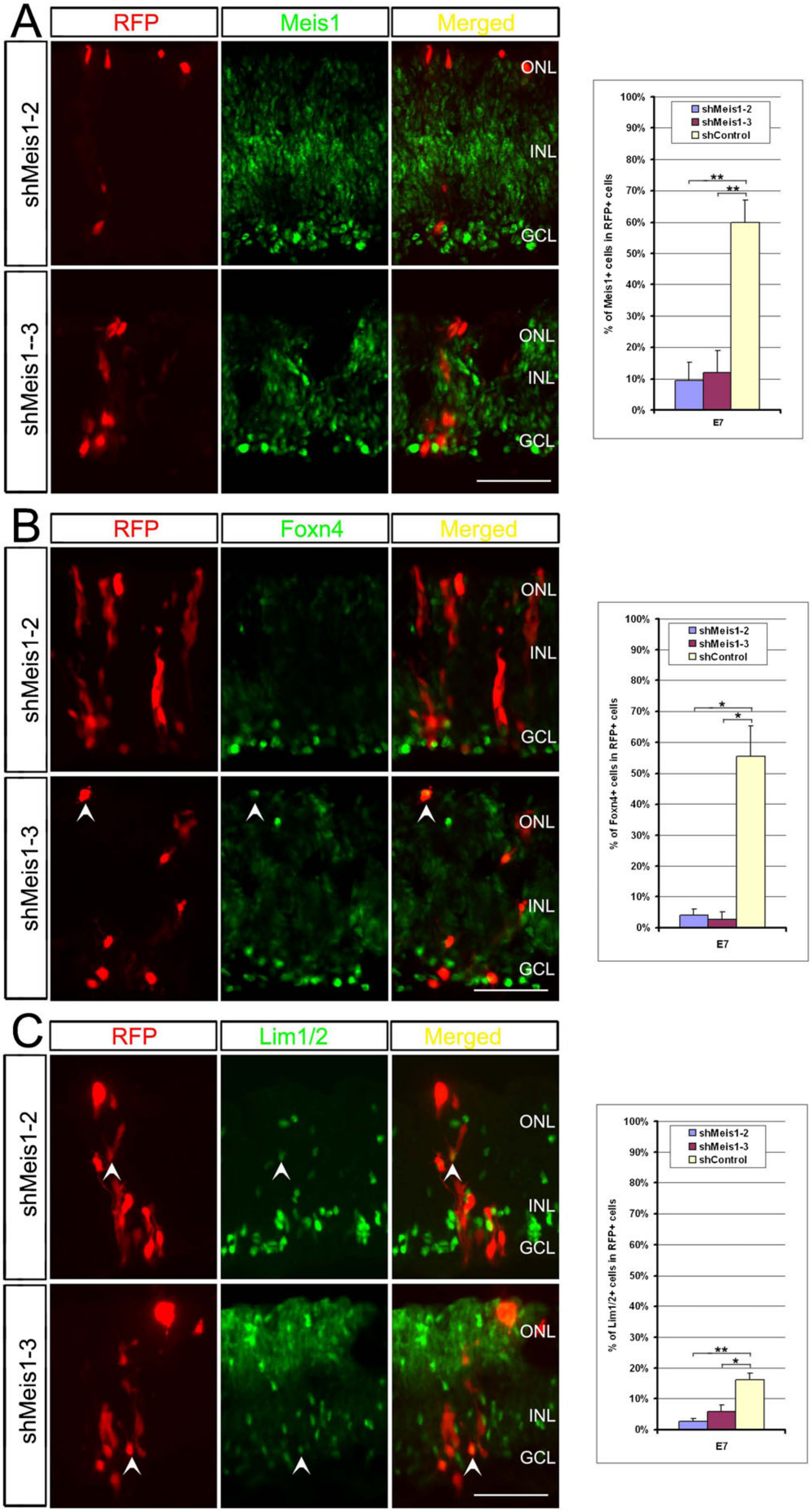

Fig. S8. Knockdown of Meis1 reduces the expression of Foxn4 and Lim1+2. Two additional Meis1-shRNA-RFP plasmids (i.e. shMeis1-2; shMeis1-3) were tested in chick retinas. Transfected retina tissues were harvested at E7 after electroporation at E4, sectioned, and immunostained with Meis1 (A), Foxn4 (B) and Lim1/2 (C). RFP+ cells resulting from shRNA transfection were observed with reduced protein level for all three markers by antibody staining (arrowheads). Histograms show that there were dramatic decreases in the percentage of Marker+/RFP+ cells in shMeis1-2 and shMeis-3 groups. The average of three independent experiments is shown; error bars indicate standard deviation. *$P<0.05$; **$P<0.005$. ONL, outer nuclear layer; INL, inner nuclear layer; GCL, ganglion cell layer. Scale bars: 50 μm.

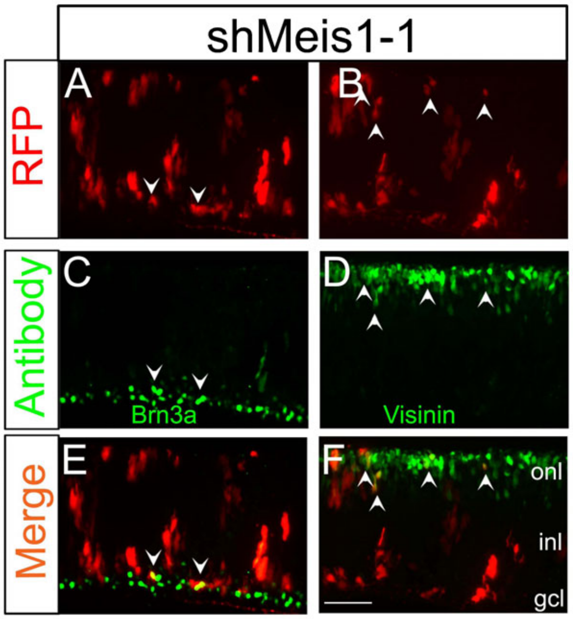

Fig. S9. Knockdown of Meis1does not affect Brn3a and Visinin expression in chick retina. Chick retinas at E7 three days after transfection with shMeis1-1 knockdown construct at E4. Transfected retinal cells (RFP+ red cells in panels A,B) were immunostained for cell specific antibodies: Brn3a and Visinin (green in panels C,D). Double labeled cells (RFP+/Brn3a+ or RFP+/Visinin+) were indicated by arrowheads (E,F). ONL, outer nuclear layer; INL, inner nuclear layer; GCL, ganglion cell layer. Scale bar: 20 µm.

Biology Open
